# Caffeine Consuming Children and Adolescents Show Altered Sleep Behavior and Deep Sleep

**DOI:** 10.3390/brainsci5040441

**Published:** 2015-10-15

**Authors:** Andrina Aepli, Salome Kurth, Noemi Tesler, Oskar G. Jenni, Reto Huber

**Affiliations:** 1 Child Development Center, University Children’s Hospital Zurich, Steinwiesstrasse 75, 8032 Zurich, Switzerland; E-Mails: aeplia@access.uzh.ch (A.A.); noemi.tesler@kispi.uzh.ch (N.T.); oskar.jenni@kispi.uzh.ch (O.G.J.); 2 Sleep and Development Laboratory, Department of Integrative Physiology, University of Colorado Boulder, 354 UCB Boulder, 80309 Boulder, CO, USA; E-Mail: salome.kurth@colorado.edu; 3 Neuroscience Center Zurich (ZNZ), Winterthurerstrasse 190, 8057 Zurich, Switzerland; 4 Zurich Center for Integrative Human Physiology (ZIHP), University of Zurich, Winterthurerstrasse 190, 8057 Zurich, Switzerland; 5 University Clinics for Child and Adolescent Psychiatry Zurich, University of Zurich, Lenggstrasse 31, 8029 Zurich, Switzerland

**Keywords:** caffeine, sleep EEG topography, development, children, adolescents, slow-wave activity

## Abstract

Caffeine is the most commonly ingested psychoactive drug worldwide with increasing consumption rates among young individuals. While caffeine leads to decreased sleep quality in adults, studies investigating how caffeine consumption affects children’s and adolescents’ sleep remain scarce. We explored the effects of regular caffeine consumption on sleep behavior and the sleep electroencephalogram (EEG) in children and adolescents (10–16 years). While later habitual bedtimes (Caffeine 23:14 ± 11.4, Controls 22:17 ± 15.4) and less time in bed were found in caffeine consumers compared to the control group (Caffeine 08:10 ± 13.3, Controls 09:03 ± 16.1), morning tiredness was unaffected. Furthermore, caffeine consumers exhibited reduced sleep EEG slow-wave activity (SWA, 1–4.5 Hz) at the beginning of the night compared to controls (20% ± 9% average reduction across all electrodes and subjects). Comparable reductions were found for alpha activity (8.25–9.75 Hz). These effects, however, disappeared in the morning hours. Our findings suggest that caffeine consumption in adolescents may lead to later bedtimes and reduced SWA, a well-established marker of sleep depth. Because deep sleep is involved in recovery processes during sleep, further research is needed to understand whether a caffeine-induced loss of sleep depth interacts with neuronal network refinement processes that occur during the sensitive period of adolescent development.

## 1. Introduction

Numerous different foods and beverages contain the alkaloid caffeine, including coffee, tea, cola, ice tea, chocolate and energy drinks, which makes caffeine the most commonly ingested psychoactive drug worldwide [1]. Caffeine is a non-specific, competitive antagonist of adenosine receptors [2]. Adenosine effects on these receptors include the up-regulation of A1 receptors and the increase of adenosine inhibition, which reduces excitatory neurotransmission, and alertness affecting sleep homeostasis [3]. The best-established marker of the homeostatic regulation of sleep is deep sleep slow-wave activity (SWA; 1–4.5 Hz) in the electroencephalogram (EEG) [4,5]. SWA is tightly coupled to prior wakefulness duration [5] and results from highly synchronized activity of cortical neurons [6]. Caffeine is considered to powerfully stimulate our brain networks during waking and sleep.

How caffeine affects sleep regulation has primarily been investigated in adults [7,8,9]. These studies show that even low doses of caffeine impact the build-up of sleep pressure by reducing SWA at the beginning of the night in adult populations. These studies also show that SWA is affected by caffeine intake that directly precedes sleep [8] or that is consumed in the morning of the same day preceding sleep [9]. Stimulant effects of caffeine in adults also include alterations in sleep continuity (*i.e.*, reduced sleep efficiency and increased sleep latency) [8,10]. While these studies show that caffeine strongly affects mature sleep brain physiology, no comparable studies exist during sensitive periods of brain development [11] (*i.e.*, in childhood or adolescence).

Caffeine consumption has significantly increased among adolescents over the past years [12]. Similar to adults, high caffeine intake in adolescents is associated with an increase of reported sleep difficulties, sleep disturbances, and morning tiredness [13,14]. One emerging question is whether high caffeine consuming children and adolescents exhibit a concomitant reduction in objective sleep quality. Furthermore, considering the growing consumption frequency of caffeine in adolescents worldwide, it is surprising that no studies have objectively quantified effects of caffeine [8,9] in children and adolescents through the gold-standard measure of sleep quality, *i.e.*, EEG activity. Addressing this gap in knowledge becomes particularly relevant given that a growing body of epidemiological literature reports early sleep difficulties as predictors for later psychological, emotional and social problems [15,16,17]. The aim of the present study was to investigate the effects of high caffeine consumption on sleep physiology and associations with sleep habits in young life.

## 2. Materials and Methods

### 2.1. Participants and Scheduling

Participants were recruited using paper and personal advertisement in schools, football clubs, boy scouts and through personal contact. Approximately 800 adolescents were handed a flyer, telephone screenings were completed with 50 adolescents, and 32 subjects were finally included in the study based on exclusion / inclusion criteria and personal interest to participate. Participants were aged 10.0 to 16.9 years, with 16 of the subjects (8 males, 11.9–16.8 years) reporting a habitual consumption of at least two servings of caffeine per day throughout the duration of at least the past three years. A standardized in-laboratory screening telephone interview was performed to obtain and evaluate habits of caffeine consumption and sleep. Caffeine consumers were contrasted with 16 healthy age- and gender-matched control subjects, who denied regular habitual caffeine intake. Although most caffeine consumers affirmed caffeine intake both in the morning and evening, no sleep difficulties were reported. For all subjects, exclusion criteria were smoking, alcohol consumption, sleep disturbances and travelling across more than two time zones within the six months prior to the assessment. The institutional review board of the cantonal ethic commission Zurich (Switzerland, Study identification number EK StV 27/07) approved the study, and the study was performed according to the Declaration of Helsinki. Written informed consent was obtained from participants and parents. Sleep assessments for females were scheduled during their follicular phase of the menstrual cycle, when sleep quality is increased compared to premenstrual and menstrual phases [18]. All sleep assessments took place at the University Children’s Hospital Zurich (Zurich, Switzerland).

### 2.2. Actigraphy, Sleep Diary and Questionnaires

During one week prior to the assessment, participants followed a stabilized bedtime schedule that was verified with wrist actigraphy and bedtime diaries. In addition, reports on foods and beverages that contain caffeine, as well as the time of consumption were obtained during this week. Subjects were advised to consume caffeine according to habitual use. Reports together with the screening questionnaires, served as quantitative measures for frequency and amount of caffeine intake. The exact caffeine content of all reported products was obtained from the manufacturers’ declarations, and caffeine content of roasted and ground coffee provided the basis for all “coffee” reports [19]. Pubertal rating was assessed with a self-administered rating scale for pubertal development, which shows reasonable internal consistency and reliability (0.67–0.70 Cronbach’s coefficient alpha) [20]. Yet pubertal rating was not used as matching variable between groups, due to age being a stronger predictor for maturational changes in the EEG than pubertal status [21]. In addition, socioeconomic status (SES) [22] and chronotype [23] were assessed. Chronotype shows good consensus with the Morning-Eveningness Questionnaire [24] and the Composite Scale of Morningness [25], and is thus a valid estimate of a subject’s circadian preference. SES quantifies school education of the mother and professional position of the father and is a marker for the family’s relative social and economic position. SES has been validated, showing adequate reliability in our community (e.g., SES correlates with intellectual and language outcome of children) [22].

In the morning following the sleep assessments, subjects rated their morning tiredness using a visual analogue scale (VAS). VAS entailed a line with endpoints marking the two extreme of the category (*i.e.*, relaxed *vs.* tired). Subjective ratings along the line were converted into a percentage value ranging from 0% to 100% for analysis.

### 2.3. Attention Task

The attention task was performed after waking up from the recording night. Subjects gazed at a white cross on a black background on a computer screen while listening to 300 regularly presented acoustic stimuli (around 80 dB, 880 Hz) during four minutes. Ten percent of these stimuli were randomly presented deviants in frequency (988 Hz). Participants were instructed to react as quickly as possible to the deviant stimuli with a mouse click. MATLAB (Mathworks, Natick, MA, USA) was used for task presentation and the calculation of the attention variable “reaction time” (time elapsed between presentation of stimulus and mouse click).

### 2.4. Sleep Assessment

Sleep assessments involved hdEEG, electromyogram (two chin electrodes) and electrooculogram, were scheduled according to individually reported habitual bedtimes, and all participants were awoken in the morning according to a scheduled wake-up time. In the caffeine group, 5 subjects were assessed on weekend nights (Friday through Sunday morning), and 11 on weekday nights, in the control group 4 assessments took place on weekend nights and 12 on weekday nights. Electrode nets with 128 channels (HydroCel Geodesic Sensor Net for long-term monitoring, EGI) were adjusted to Cz and mastoids and filled with gel electrolyte, which ensures maintenance of good quality signals even after several hours of recording. Impedance level was set below 70 kΩ and the signal was referenced to Cz for direct visualization (re-references to the average across all channels, details follow) with a sampling rate of 500 Hz, and then off-line bandpass filtered (0.5–50 Hz), and down sampled to 128 Hz. Sleep stages were visually scored in 20 s epochs according to standard criteria [26]. Semi-automatic artefact correction was performed based on two frequency bands (0.75–4.5 Hz and 20–30 Hz, see [27]). As in previous studies [28,29] only the 109 derivations located above the ears were included for analysis (to avoid artifacts via facial and neck muscles) and all derivations were re-referenced to the average across all electrodes. Using a standard approach [28,30] a fast Fourier transform routine was performed (FFT, Hamming window, average of five 4-s epochs), log-transformed average power spectra (resolution of 0.25-Hz-bins) were calculated for group comparisons, including non-rapid eye movement sleep (NREM) sleep stages 2 and 3. Analyses were performed using MATLAB (Mathworks, Natick, MA, USA) and SPSS (IBM). For topography analysis, EEG power for each electrode was plotted on the planar projection of the scalp using the topoplot function of the MATLAB EEGLAB toolbox [31].

### 2.5. Statistics

We verified normality of data distribution using nonparametric Kolmogorov-Smirnov test for one sample (comparison to an expected distribution), and homoscedasticity was examined with Levene’s test. For group comparisons, two-tailed, unpaired Student’s *t*-tests (for normally distributed data) or Mann-Whitney-U tests (for not normally distributed data) were calculated. Where homoscedasticity was not met, the *p*-value corrected for lack of homogeneity of variance is included. We corrected for multiple comparisons using false discovery rate (FDR). For power analysis, group means were compared using a two-sided equality test. An effect size of 80% and an alpha level of 0.05 was chosen. Significant topographical differences were assessed by statistical nonparametric mapping (SnPM), using single threshold tests [32]. As in our previous work [33,34], we calculated *t-*values for each electrode, found the maximal t-value across all electrodes for each permutation, and determined the *t*-value threshold as the 95th percentile of the permutation-derived *t*-values. Electrodes exceeding this threshold were considered significant regarding topographical group differences.

## 3. Results

### 3.1. Demographic Characteristics

Caffeine consumers and controls did not differ in age, weight, Tanner stage, chronotype, or SES (Table 1). As expected, caffeine intake per day as well as caffeine intake controlled for body weight differed clearly between caffeine consumers and controls.

**Table 1:** Demographic characteristics of the caffeine consumers and the controls.

	Caffeine Consumers (*n* = 16)	Controls (*n* = 16)
Age (years); [f, m]	14.5 ± 0.4 (11.9–16.8); [15.1 ± 0.6, 14.0 ± 0.4]	14.4 ± 0.4 (11.8–16.4); [15.0 ± 0.5, 13.8 ± 0.5]
Caffeine consumption (mg/kg/d)	2.5 ± 0.4 *	0.1 ± 0.0 *
Chronotype	4.3 ± 0.2	3.8 ± 0.2
Socio Economic Status, SES	5.0 ± 0.5^(5)^	3.8 ± 0.3^(7)^
Sex (f, m)	8 f, 8 m	8 f, 8 m
Tanner puberty scale; [f, m]	7.7 ± 0.7^(3)^; [8.4 ± 1.3, 7.9 ± 0.9]	7.5 ± 0.6^(2)^; [7.7 ± 1.1, 7.4 ± 0.7]
Weight (kg)	52.3 ± 2.6	51.2 ± 3.7^(6)^

Table 1, reports scores (M ± SEM) for the caffeine consumers and the controls. Number (n) of subjects missing is indicated with footnotes (n). (f = female; m = male). Significant differences are indicated with asterisk (* *p* < 0.05, two tailed, unpaired Student’s *t*-test).

### 3.2. Sleep Diary, Architecture, Subjective Records and Attention Task

Sleep diaries showed that caffeine consumers went to bed 57 min later (Caffeine 23:14 ± 11 [clock time ± min], Control 22:17 ± 15) and spent 53 min less time in bed (Caffeine 08 h 10 min ± 13 min, Control 09 h 03 min ± 16 min) compared to the control group (Table 2). The difference of time in bed between weekend-days (nights from Friday to Saturday and from Saturday to Sunday) and week-days did not vary between groups (Caffeine 01 h 03 min ± 19 min, Control 01 h 18 min ± 21 min). The time of latest caffeine consumption was reported as 16:37 ± 00:46 (average across days). Participants slept well during the recording night (> 89% average sleep efficiency in both groups, Table 2) and showed no group difference in sleep architecture (Table 2). Neither subjectively reported tiredness in the morning nor an objective measure of tiredness (reaction time in an attention task) differed between groups (Table 2). No relationship was found between morning tiredness and Tanner stage.

**Table 2:** Sleep diary, architecture, continuity, subjective reports and attention task.

Data	Point in time	Sleep Variables	Caffeine Consumers (*n* = 16)	Controls (*n* = 16)	Estimated *n* to Reveal Group Difference
Diary	*Week prior*	Rise time (hrs:min)	07:29 ± 7.6	07:20 ± 7.5 °	170
		Bed time (Lights out) (hrs:min)	23:14 ± 11.4 *	22:17 ± 15.4 °	13
		Time in bed entire week (hrs:min)	08:10 ± 0:13 *	09:03 ± 0:16 °	18
		Time in bed week days (hrs:min)	08:07 ± 0:19	08:28 ± 0:10	120
		Time in bed weekend days (hrs:min)	09:04 ± 0:16	09:42 ± 0:27	81
		Difference time in bed weekend-days and time in bed weekdays (hrs:min)	01:03 ± 0:19	01:18 ± 0:21	342
		Last caffeine consumption (hrs:min)	16:37 ± 00:46	n.a.	n.a.
Sleep architecture and continuity (EEG)	*Night of assessment*	Time in bed (hrs:min)	07:48 ± 15.4	08:10 ± 16.7	157
		Total sleep time (hrs:min)	06:59 ± 0:15	07:39 ± 0:18	44
		Sleep onset latency (min)	20.3 ± 4.1	15.8 ± 1.8	108
		Wakefulness after sleep onset (min)	32.1 ± 6.7	17.6 ± 2.5	26
		Stage 1 (% of total sleep time)	6.8 ± 1.3	5.8 ± 0.6	221
		Stage 2 (% of total sleep time)	48.6 ± 1.4	49.1 ± 1.9	2689
		Slow-wave sleep (% of total sleep time)	27.2 ± 2.0	24.1 ± 1.8	91
		REM sleep (% of total sleep time)	17.5 ± 1.0	20.2 ± 1.7	62
		Sleep efficiency (total sleep time/time in bed) (%)	89.6 ± 1.9	93.7 ± 0.6	24
Subjective reports/attention task	*Morning after assessment*	Reaction time in attention task (ms)	283.6 ± 8.4 °°	287.9 ± 7.9 °°	733
		Subjectively reported morning tiredness (relaxed *vs.* tired) (%)	51.9 ± 3.4	53.7 ± 5.8	1637

° *n* = 14; °° *n* = 13.

Table 2, Sleep parameters are indicated as M ± SEM, sleep architecture was taken from visual scoring and relative values are calculated for each subject separately and then averaged for groups. Diaries and reports about the last caffeine consumption refer to reports during the week prior to the assessments. In the EEG, sleep onset latency refers to the first occurrence of stage 2 sleep. Subjective statements about morning tiredness were reported directly after the recording session. Reaction time in the attention task was averaged across all behavioral responses. Significant group differences are indicated with asterisk (* *p* < 0.05; two-tailed, unpaired Student’s *t*-test). Power analysis indicates minimal group size required to obtain a significant result (comparison of group means, 2-sided equality test, effect size 80%, alpha level 0.05).

### 3.3. EEG Power

**Figure 1:**
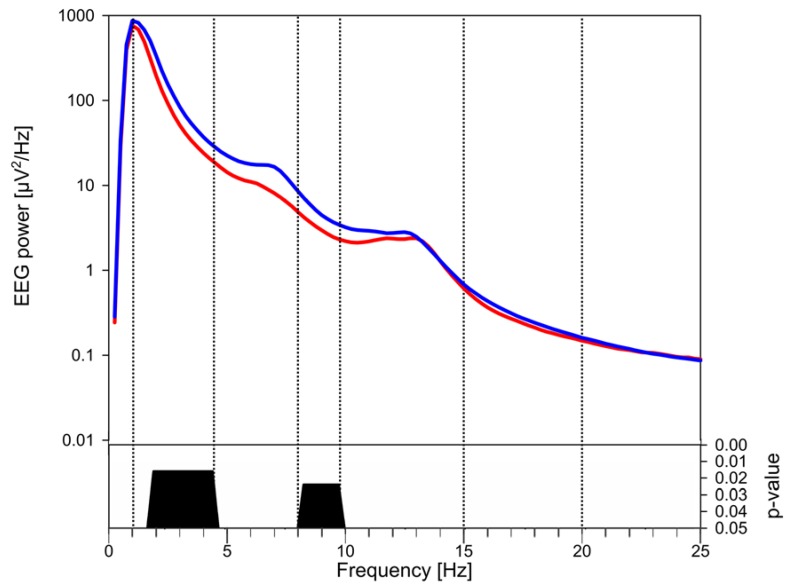
Power spectrum of the sleep electroencephalogram (EEG) in caffeine consumers (red, *n* = 16) and controls (blue, *n* = 16). Group-wise comparisons were performed with the first two hours of NREM sleep EEG data using the average across all 109 electrode channels for each subject within each frequency bin within 0.25 and 25 Hz. Commonly used EEG frequencies are separated by vertical lines: SWA = 1–4.5 Hz; theta = 4.75–7.75Hz; alpha = 8–9.75 Hz; beta = 20–25 Hz. Frequency bins (0.25-Hz) within which power differed significantly (*p* < 0.05; two-tailed, unpaired Student’s *t*-test) and the level of their significance are shown in the lower panel, only significant p values (after FDR correction) are plotted.

**Figure 2:**
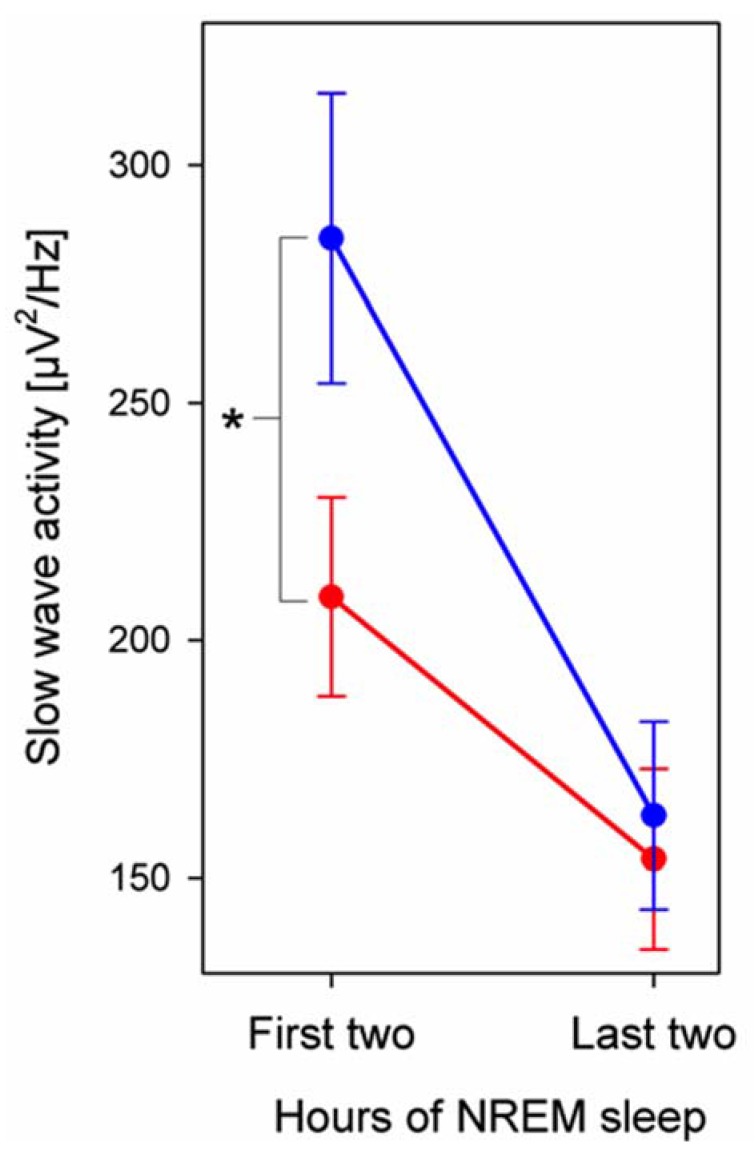
Slow-wave activity (SWA) across the night in caffeine consumers (red, *n* = 16) and controls (blue, *n* = 16). SWA (1–4.5 Hz) was averaged across all electrodes (M ± SEM) for the first and last two hours (latest possible artifact-free, common two hours among all subjects) of NREM sleep stages N2–3. The asterisk indicates a significant difference between caffeine consumers and controls in the first two hours of NREM sleep (*p* < 0.05, two-tailed, unpaired Student’s *t*-test).

In the first 2 h of NREM sleep absolute EEG power was lower in caffeine consumers compared to control subjects, encompassing large frequency bands within SWA (1–4.5 Hz), and alpha (8.25–9.75; Figure 1). We calculated SWA across the night to investigate the persistence of this effect and found that group differences disappeared by the morning (Figure 2). This EEG power convergence also occurred in alpha activity. Because overall group differences were most prominent in early sleep and low frequencies, we then focused on the SWA frequency range in the first two hours of NREM sleep for further analyses.

**Figure 3:**
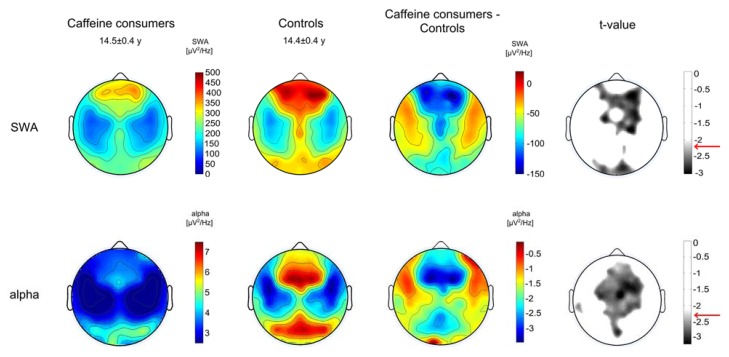
Regional sleep slow-wave activity (SWA, 1–4.5 Hz) and alpha activity (8.25–9.75) in caffeine consumers (*n* = 16) and controls (*n* = 16). Upper panels show the topographical distribution of SWA averaged for the two groups in NREM stages 2 and 3 during the first two hours of NREM sleep, group differences and the level of significance (*t* < −2.21, two-tailed, unpaired *t*-test). Lower panels show the topographical distribution of alpha activity averaged for the two groups in NREM stages 2 and 3 during the first two hours of NREM sleep, group differences and the level of significance (*t* < −2.25, two-tailed, unpaired *t*-test). The significance level is indicated with a red arrow. Values are color coded (maxima in red, minima in blue, respectively maxima in white, minima in black for *t*-values) and plotted on the planar projection of a hemispheric scalp model. Maps for caffeine consumers and controls are presented using the same color scale, and statistical maps are proportionally scaled to optimize color contrast. Values between electrodes were interpolated in each plot.

SWA topography appeared fairly symmetrical between hemispheres for both groups (Figure 3) with local maxima and minima in line with previous studies, *e.g.*, [28]. Group differences prevailed over wide areas, showing lower SWA in caffeine consumers: SWA was significantly reduced in 33% of all electrodes with an average reduction of 20% ± 9% across all electrodes and subjects. Largest effects predominated over prefrontal, central and occipital regions (Figure 3). Topographical comparisons of alpha activity also revealed group differences that were pronounced in similar areas, such that caffeine consumers exhibited reduced power over prefrontal, central, parietal and occipital regions (Figure 3).

## 4. Discussion

We report effects of regular caffeine consumption on sleep behavior and brain activity during sleep in children and adolescents. In particular, we examined whether caffeine consumption is associated with altered sleep habits and brain activity during children’s and adolescents’ sleep as reported in adults [8,9]. First, our findings show that regular caffeine consumption in young subjects is associated with later bedtimes and shorter time in bed. Second, caffeine-consuming subjects reveal reduced SWA and alpha activity with strongest effects over prefrontal, central and occipital regions. Finally, the SWA reduction appears mostly at the beginning of the night, which is consistent with findings in adults [8,9]. Our results suggest that only minor changes occur in sleep architecture (*i.e.*, sleep stages) and continuity and that regular caffeine consumption in adolescents may lead to later bedtimes and reduced sleep depth, as measured by SWA.

In adults, caffeine attenuates the build-up of sleep SWA during the day [8,9] and thus reduces sleepiness. We consequently expected later bedtimes in young caffeine consumers, which was confirmed with the sleep diary data. Diaries were shown to be a tool equally reliable to actigraphy for the assessment of sleep (bedtimes, sleep start [35]). At what time of the day caffeine was consumed was variable across subjects (*e.g.*, see variability of latest caffeine intake, Table 2), which may have contributed to individual differences in the effects of caffeine. Because habitual rise times did not differ between groups, the question arises whether caffeine consumers are chronically sleep restricted, which would increase tiredness and other symptoms associated with sleep loss, such as impairment of working memory or inhibitory control [36,37,38]. We assumed that subjective statements about morning tiredness are a reliable inverse measure for being well rested, and similarly a short reaction time in the attention task. It is interesting that these measures did not differ between groups, which may imply that daily functioning is not affected by caffeine consumption. However, as our power analysis indicates (Table 2), larger samples may reveal additional group differences. It is conceivable that the shortened time spent in bed may increase caffeine intake throughout the subsequent day to combat daytime sleepiness [39]. Other studies, however, have reported increased morning tiredness in high caffeine consuming adolescents, compared to controls [13]. However, caffeine consumption in terms of exact volume has not been computed in that study and a different quantification of subjective tiredness was used, a caveat when directly comparing outcomes between studies. It will be relevant in future studies to track daytime sleepiness throughout the day and examine interactions between sleepiness and acute caffeine intake. On the other hand, if caffeine consumers were chronically sleep deprived, they might have underestimated subjective tiredness, because sleep restricted adults are largely unaware of increasing cognitive deficits [37].

Alpha activity revealed topographical effects similar to SWA when contrasting caffeine consumers with the control group. A close relationship between alpha and SWA is an often-observed phenomenon (*i.e.*, “alpha-delta”) that is characterized as alpha waves superimposed on slow waves [40,41]. The alpha-delta relationship has been discussed in the context of the perception of sleep depth and the estimation of sleep time, however with controversy in the literature [40]. Our finding that alpha and SWA group differences were topographically similar further supports the alpha-delta link, but it remains to be investigated whether reduced alpha power in caffeine consumers accounts for perceived sleep quality and duration in children and adolescents.

The stimulatory effect of caffeine is central. The action of the adenosine antagonist affects mainly sleep quality and the cardiovascular system [1]. By doing so, caffeine modulates the cAMP signaling pathway [2], which plays a key role in neural function [42]. Adenosine accumulation, on the other hand, was suggested to prevent neurodegeneration [43]. It is thus likely that caffeine affects neuronal plasticity. Numerous studies illustrate the importance of sleep SWA for neuronal plasticity [44,45] and the caffeine induced reduction of SWA may affect synaptic plasticity during cortical maturation. Alternative pathways of action are also conceivable: Caffeine may impact plasticity via microglia, which play a key role in synaptic pruning [46]. Chronic caffeine ingestion impacts microglia morphology and density [47]. A further potential interference of caffeine with synaptic plasticity might originate from the blocked activation of the actin binding protein Cofilin, which is central for synaptic plasticity mechanisms [48]. No matter what mechanisms underlie the impact of caffeine on plasticity, further research is needed to understand whether and how caffeine consumption throughout the critical period of adolescence may ultimately alter cortical maturation.

Finally, our findings are also of practical relevance in the on-going discussion about adjusting school start times to adolescent’s needs [49]. Given that rise times are determined by school schedules, later school start times may allow caffeine consumers to be comparably well rested through compensating for later bedtimes and potentially worse sleep quality (increased subjectively reported and objectively measured wakefulness after sleep onset). Conversely, one problem of this view is the fact that caffeine consumers do not seem to compensate for reduced time in bed with increased sleep duration at weekends (no difference of time in bed between week days and weekend days). Thus, sleep need might generally be reduced in caffeine consuming children and adolescents. Additional support for this hypothesis comes from our finding that caffeine consumers exhibit reduced SWA, the best-established physiological marker of sleep need [50]. SWA levels at the beginning of the night were lower in caffeine consumers compared to controls, but converge to equivalence in the morning hours. This convergence is in line with the subjective perception of groups being equally rested (no group difference in “tiredness”). However, considering that sleep is involved in plastic processes of neural networks [51], it remains to be examined whether such an adaptive response to caffeine consumption that results in reduced sleep, affects development. Alternatively, we cannot exclude the possibility that children and adolescents with less deep sleep and a lifestyle involving later bedtimes have more social demands and are more prone to consume high amounts of caffeine [52]. These data are critical for gaining insight into the possible vulnerability of the developing brain through extensive caffeine consumption. Additionally, it is possible that caffeine consumers exhibit a reduced capacity for the state of deep sleep related to cortical hyperarousal, which can be induced through caffeine [53]. Yet, we did not find group differences in wake after sleep onset, nor did we find increased beta activity, which was proposed to reflect a cortical hyperarousal in the context of insomnia [54]. However, these findings need to be interpreted in the context of limitations. The cross-sectional nature of the data and the rather small sample size does not allow developmental conclusions and limits the statistical power of our study. Further, the nature of the study design does not allow to address causality, and prospective examinations are required that investigate mediators that may be driving the correlation between caffeine consumption and sleep (*e.g.*, social context, lifestyle, time point of caffeine intake). However, because no correlative relationship was found between bedtime and the individual extent of SWA, it is unlikely that bedtime has caused the effect of reduced SWA in a homeostatic manner.

## 5. Conclusions

In summary, this study shows that regular caffeine consumption in children and adolescents is associated with later bedtime and affects brain activity during sleep similar to adults. Because childhood and adolescence are sensitive periods of neural network refinement processes, where sleep may play a prominent role [53], longitudinal and experimental studies will provide clarity on whether intensive caffeine consumption can alter neurodevelopmental trajectories as reported in animals [55].
